# Baseline gut microbiome features associated with fecal calprotectin response to exclusive enteral nutrition in pediatric Crohn’s disease

**DOI:** 10.3389/fped.2026.1833193

**Published:** 2026-06-08

**Authors:** Hongwei Zheng, Wenbiao Chen

**Affiliations:** School of Medicine, Quanzhou Medical College, Quanzhou, Fujian, China

**Keywords:** 16S rRNA gene sequencing, exclusive enteral nutrition, fecal calprotectin, gut microbiome, pediatric Crohn's disease

## Abstract

**Background and aims:**

Exclusive enteral nutrition is an established induction therapy for pediatric Crohn's disease, but biochemical response varies across patients. We assessed whether baseline gut microbiome features were associated with fecal calprotectin response and whether treatment-related taxonomic shifts could be interpreted alongside reported fecal biochemical summaries.

**Methods:**

We re-analyzed a public pediatric cohort (PRJEB14084). Paired baseline and end-of-treatment microbiome profiles were summarized as within-subject genus-level changes. Reported stool biochemical summaries were used as contextual data. Baseline model performance was explored by repeated nested cross-validation with calibration assessment. An independent pediatric cohort (PRJEB33603) was used only for summary-level external comparison.

**Results:**

Several genera changed consistently during treatment. Stool biochemical summaries showed lower total amino acids and tryptophan and higher secondary bile-acid proportion and hydrophobicity index at the end of treatment. The baseline model showed modest discrimination with broad uncertainty (mean area under the receiver operating characteristic curve, 0.655; 95% confidence interval, 0.450–0.850). In the external comparison, 7 of 12 overlapping genera showed concordant directions.

**Conclusion:**

In this public cohort, baseline microbiome features showed an exploratory association with fecal calprotectin response, but the estimates were imprecise. External comparison was limited to published summary tables, and all inferences remain association-based.

## GRAPHICAL ABSTRACT

**Figure:**
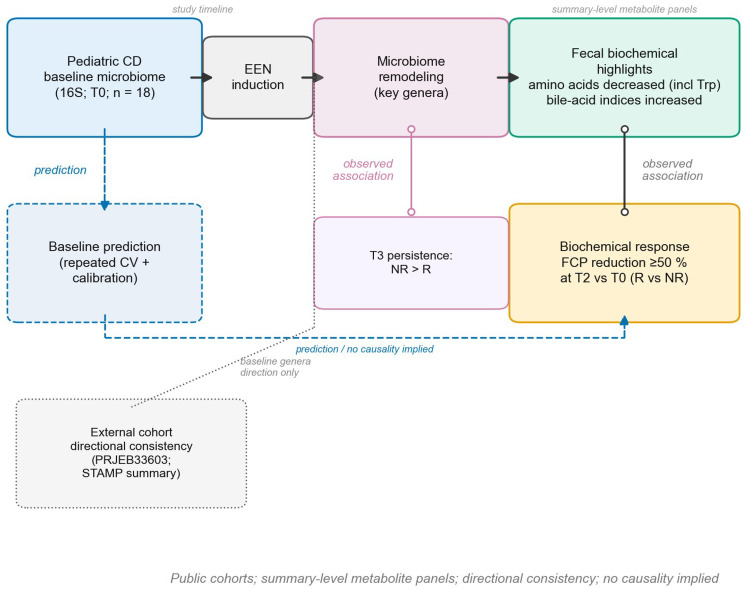
Schematic overview of the study design, summarizing baseline microbiome–response association, longitudinal microbiome remodeling during exclusive enteral nutrition, biochemical context from reported summary panels, and an external summary comparison based on published tables. The schematic is association-focused and does not imply causality. Source Data: not applicable (schematic).

## Introduction

Crohn's disease (CD) frequently presents during childhood and adolescence and may have lifelong consequences for growth, nutritional status, and quality of life. Exclusive enteral nutrition (EEN) is a first-line induction therapy for pediatric CD and can improve both clinical and biochemical outcomes without the systemic adverse effects associated with corticosteroids. However, treatment response is heterogeneous, and clinically useful biomarkers are needed to help stratify likely responders, guide early escalation, and clarify how dietary intervention reshapes the intestinal ecosystem (1–9).

The gut microbiome has been repeatedly implicated in pediatric CD pathophysiology and treatment response (10–13). Diet is a major determinant of microbial composition and metabolism, and EEN provides a controlled nutritional intervention through which the microbiome–host interface can be examined. Beyond taxonomy, the biological effects of EEN are mediated within a small-molecule milieu that includes amino-acid availability, bile-acid composition, and other metabolites relevant to epithelial barrier integrity and mucosal immune signaling. However, public cohorts often lack per-sample metabolite resolution and may provide only summary-level outputs, limiting participant-level multi-omics integration.

Fecal calprotectin (FCP) is widely used as a non-invasive marker of intestinal inflammation and is commonly used to monitor biochemical response in pediatric inflammatory bowel disease. In PRJEB14084, responders were defined in the source publication as having a more than 50% reduction in FCP at the end of EEN (T2) relative to baseline (T0) (1). We adopted this pre-specified responder definition to remain consistent with the original cohort study and to avoid *post hoc* threshold selection.

In this secondary analysis, we used PRJEB14084 as the primary cohort to quantify genus-level remodeling during EEN, place these taxonomic shifts in the context of reported stool biochemical panels, and examine whether baseline microbiome features were associated with biochemical response. We also compared genus-level effect directions with published summary tables from an independent pediatric EEN cohort (PRJEB33603). Because both biomolecule data and external comparisons were available only at summary level, our aim was to generate clinically relevant hypotheses while keeping interpretation within clear observational limits (14–20).

Compared with the primary publications, this secondary analysis adds three practical elements: clearer links between each figure and its supporting tables (Source Data 1–9, archived in Zenodo), explicit reporting of uncertainty and calibration for baseline association models in a small-cohort setting (rather than discrimination alone), and an external comparison limited to genus-level direction concordance without implying patient-level validation.

## Materials and methods

### Study design, cohorts, and endpoints

This study re-analyzed publicly available data from a pediatric Crohn's disease cohort treated with exclusive enteral nutrition (EEN) (PRJEB14084) and an independent pediatric EEN cohort used for summary-level external comparison (PRJEB33603). For PRJEB14084, timepoints were harmonized as baseline (T0), end of EEN (T2; approximately 6 weeks), and follow-up after return to habitual diet (T3), as defined in the source publication. Biochemical response was defined at the subject level as a fecal calprotectin (FCP) reduction of at least 50% at T2 relative to T0 (Table 1).

**Table 1:** Cohorts and analytic sample sizes used in this study.

Cohort (ENA)	Evidence used here	Key sample sizes and limitations
PRJEB14084 (discovery)	16S OTU table (OTU/genus); HPLC fecal amino-acid and bile-acid panels (summary); 1H-NMR discriminant summary (summary)	Baseline 16S: *n* = 27. Paired T0–T2 16S (all participants): *n* = 19 subjects. Baseline association model (binary FCP responder status at T2 vs. T0): *n* = 18 baseline samples (responders *n* = 8; non-responders *n* = 10). Follow-up 16S at T3: *n* = 13 (responders *n* = 5; non-responders *n* = 8). Metabolite data were available only in summary form; no participant-level microbiome–metabolite coupling or causal inference is intended.
PRJEB33603 (external)	Published STAMP differential taxa summary tables	Summary-level external comparison only; supports genus-level direction comparison, not patient-level validation.

In PRJEB14084, EEN was administered for 6 weeks followed by a 2-week taper, and the follow-up timepoint T3 corresponds to approximately 4 ± 1 months after EEN initiation in the source publication (1). Because participant-level follow-up timing was not available in the public tables we re-analyzed, we report T3 as a harmonized follow-up label rather than as an exact per-participant interval.

### Microbiome data sources and preprocessing

For PRJEB14084, we used the author-provided OTU count matrix and taxonomy annotations from the linked Supplementary Materials to ensure consistency with the original processing. OTU counts were aggregated to the genus level following taxonomic nomenclature standardization, and relative abundances were derived via total sum scaling (TSS).

For baseline model analyses, count matrices (OTU or genus) were transformed using a centered log-ratio (CLR) transform with a pseudocount of 1. Genus-level results are reported as descriptive abundance shifts and should be interpreted as compositional (i.e., relative) changes (16).

For clarity, the source tables underlying the figures are archived in Zenodo (https://doi.org/10.5281/zenodo.18815000) and provided as Supplementary Source Data. A Supplementary map table links each “Source Data” label cited in the Results and figure legends to the corresponding tab-separated tables.

### Longitudinal genus changes during EEN

**Figure 1:**
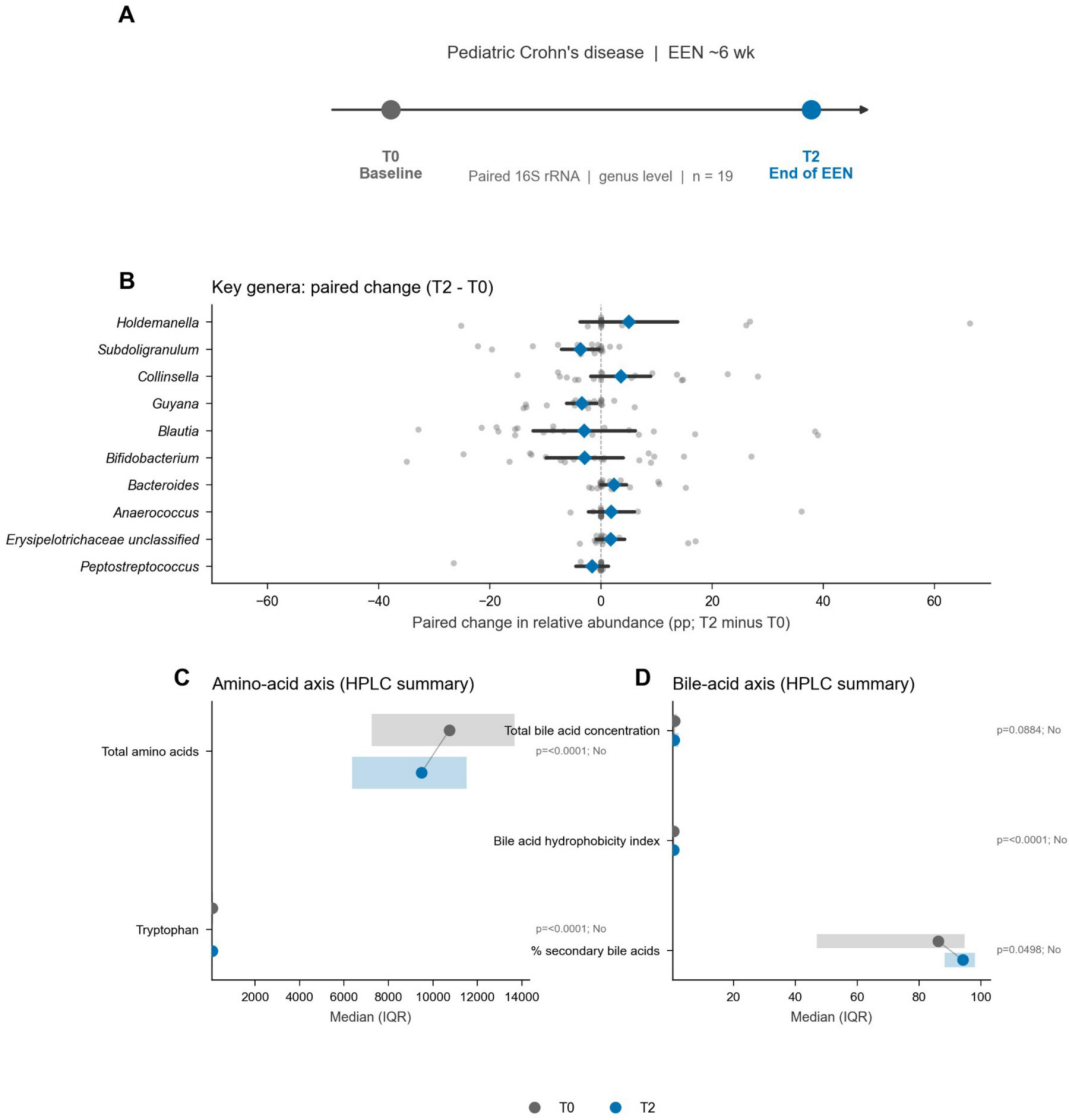
Key gut genera remodeling and measured fecal biomolecule panel shifts during exclusive enteral nutrition in pediatric Crohn’s disease. **(A)** Study timeline and timepoint definitions at baseline and end of treatment (approximately 6 weeks). **(B)** Genus-level 16S ribosomal RNA gene profiles were summarized as within-subject paired changes for participants with both timepoints available (*n* = 19; responders and non-responders combined). Key genera are shown with individual participant changes, means, and 95% confidence intervals. **(C)** The targeted fecal amino-acid panel measured by high-performance liquid chromatography is shown for total amino acids and tryptophan as median and interquartile range at baseline and end of treatment (as reported: baseline *n* = 43; end-of-treatment *n* = 22). Statistical annotations were extracted from the source publication Supplementary Tables (Supplementary Tables S7–S9 in MOESM2 ESM) (1). **(D)** The fecal bile-acid panel measured by high-performance liquid chromatography is shown as median and interquartile range at baseline and end of treatment. Statistical annotations were extracted from the source publication supplementary tables (Supplementary Tables S10–S12 in MOESM2 ESM) (1). Because paired participant-level metabolite concentrations were not available, these panels provide measured biochemical context rather than participant-level coupling tests. Source Data: Source Data 1–2.

Within-subject paired changes were computed for subjects with both baseline (T0) and end-of-EEN (T2) 16S samples available. For each genus, we calculated the mean paired change (T2 minus T0) in relative abundance and a 95% confidence interval for the mean using a t-distribution. Key genera were selected as the top-ranked genera by absolute mean paired change among genera with sufficient paired observations (Figure 1).

### Follow-up response-stratified taxa comparisons

**Figure 2:**
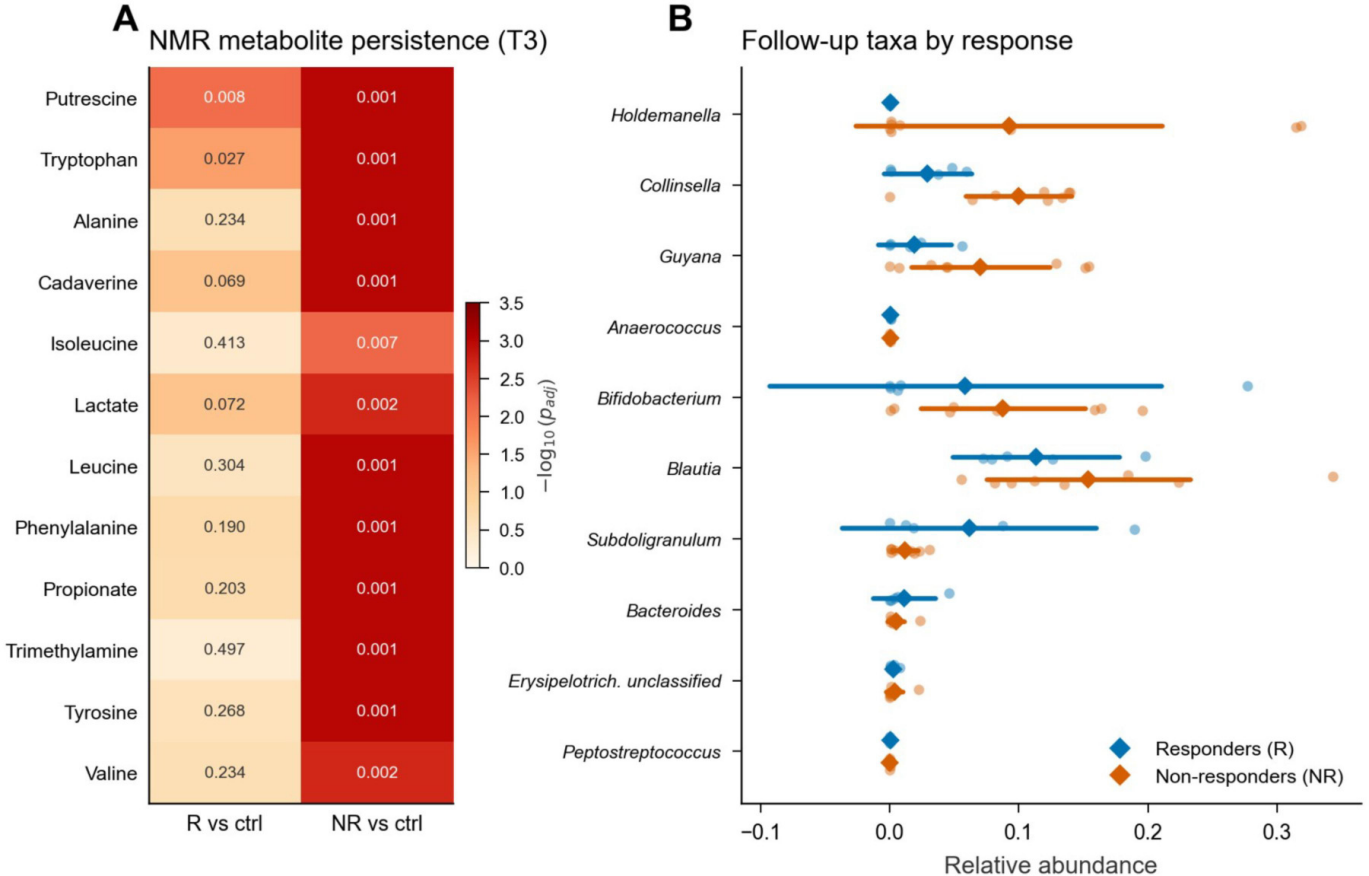
Response-linked persistence of a fecal biochemical signature at follow-up supported by discriminant metabolite summaries and follow-up taxa distributions. **(A)** Proton nuclear magnetic resonance discriminant metabolite summary at follow-up after return to habitual diet compares responders with controls and non-responders with controls using Benjamini-Hochberg adjusted *p*-values as reported in the source publication supplement (Supplementary Table 6 in MOESM2 ESM) (as reported: responders, *n* = 7; non-responders, *n* = 10; controls, *n* = 31) (1). This panel summarizes persistence and normalization patterns rather than metabolite direction of change because only discriminant summaries are available. **(B)** Follow-up genus-level relative abundance distributions compare responders (*n* = 5) and non-responders (*n* = 8) for the same key genera highlighted in Figure 1; individual values and group summaries are both shown. Source Data: Source Data 3–4.

At follow-up (T3), genus relative abundances were summarized separately in responders and non-responders. Between-group comparisons used a Wilcoxon rank-sum test, and results are presented as descriptive alignment with response-linked persistence patterns observed in the fecal biochemical signature (Figure 2).

### Biomolecule panels (HPLC and 1H-NMR)

Targeted fecal metabolite panels measured by HPLC and discriminant metabolite summaries reported by 1H-NMR were extracted from Supplementary Tables associated with PRJEB14084. HPLC data were available only as summary statistics (medians and IQRs) with reported *p*-values; therefore, analyses were restricted to reporting measured directional context rather than participant-level coupling between taxa and metabolites. For follow-up 1H-NMR data, BH-adjusted *p*-values for responder/non-responder contrasts vs. healthy controls were used to summarize persistence and normalization patterns.

### Baseline model analysis and calibration

**Figure 3:**
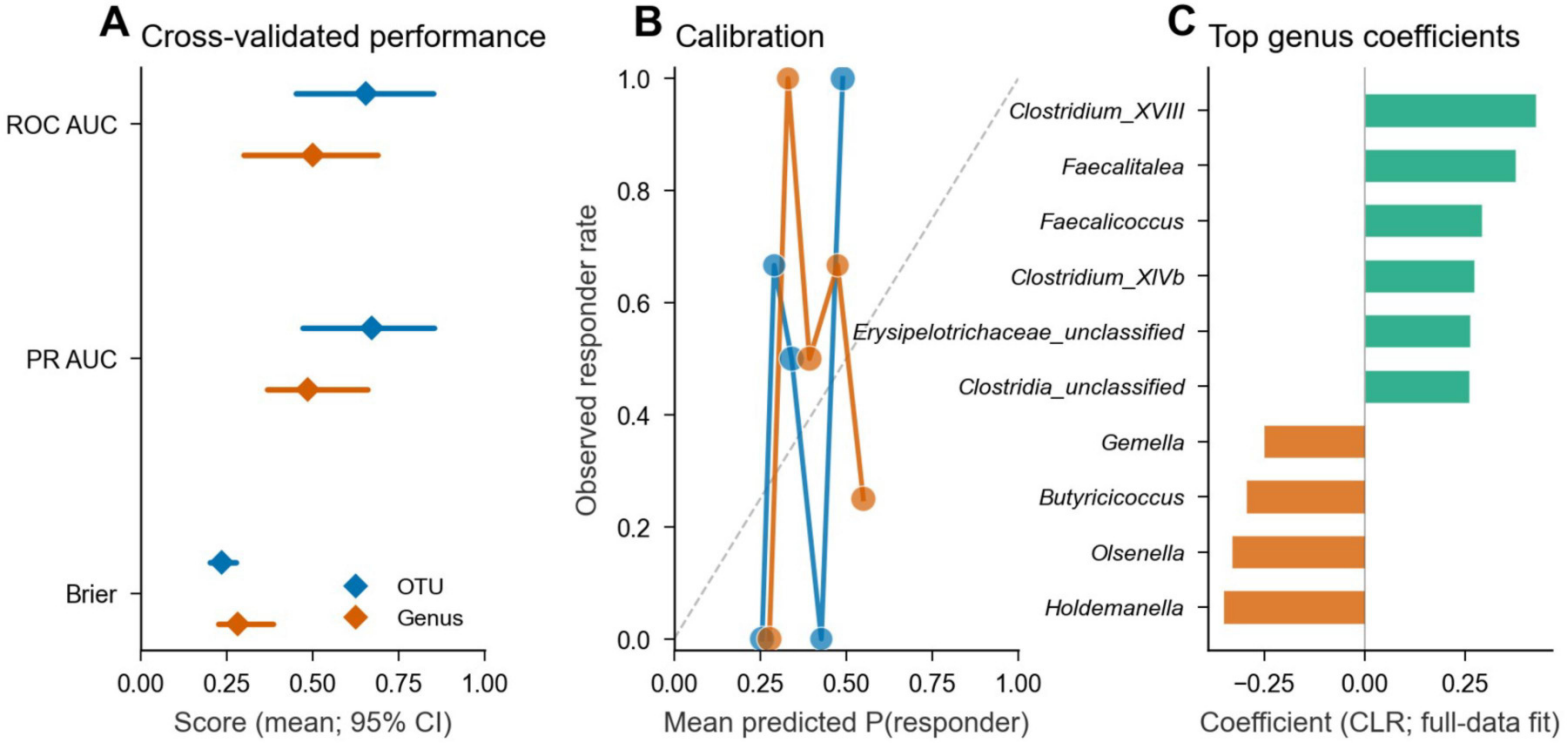
Exploratory baseline discrimination of biochemical response to exclusive enteral nutrition with uncertainty and calibration in cohort PRJEB14084. **(A)** Performance summary from repeated nested cross-validation on baseline samples (*n* = 18), reporting means and 95% confidence intervals for area under the receiver operating characteristic curve, area under the precision-recall curve, and Brier score for operational taxonomic unit-level and genus-level centered log-ratio features. **(B)** Calibration plot using mean held-out predicted probabilities per participant, summarized into five quantile bins, with a diagonal reference for perfect calibration. **(C)** Exploratory interpretability panel showing the top genus coefficients from a model fit to the full dataset; coefficients indicate association direction and are presented for hypothesis generation only. Source Data: Source Data 5–7.

We trained logistic regression classifiers using baseline microbiome composition to examine association with biochemical response (FCP reduction of at least 50% at T2 relative to T0). Model performance was evaluated using repeated nested cross-validation (200 repetitions) with stratified 3-fold splits for tuning and assessment (Table 2). Regularization strength was tuned over a prespecified grid by maximizing mean ROC AUC. Predictions from held-out folds were used to estimate ROC AUC, PR AUC, and Brier score. Calibration was assessed using mean cross-validated predicted probabilities per participant, summarized into quantile bins (18–20) (Figure 3).

**Table 2:** Baseline model performance in PRJEB14084 (repeated nested cross-validation; *n* = 18 baseline samples; 200 repeats).

Feature level	Mean ROC AUC (95% CI)	Mean PR AUC (95% CI)	Mean Brier score (95% CI)
OTU	0.655 (0.450–0.850)	0.671 (0.471–0.854)	0.237 (0.202–0.278)
Genus	0.500 (0.300–0.688)	0.485 (0.368–0.659)	0.281 (0.227–0.385)

### External summary comparison

**Figure 4:**
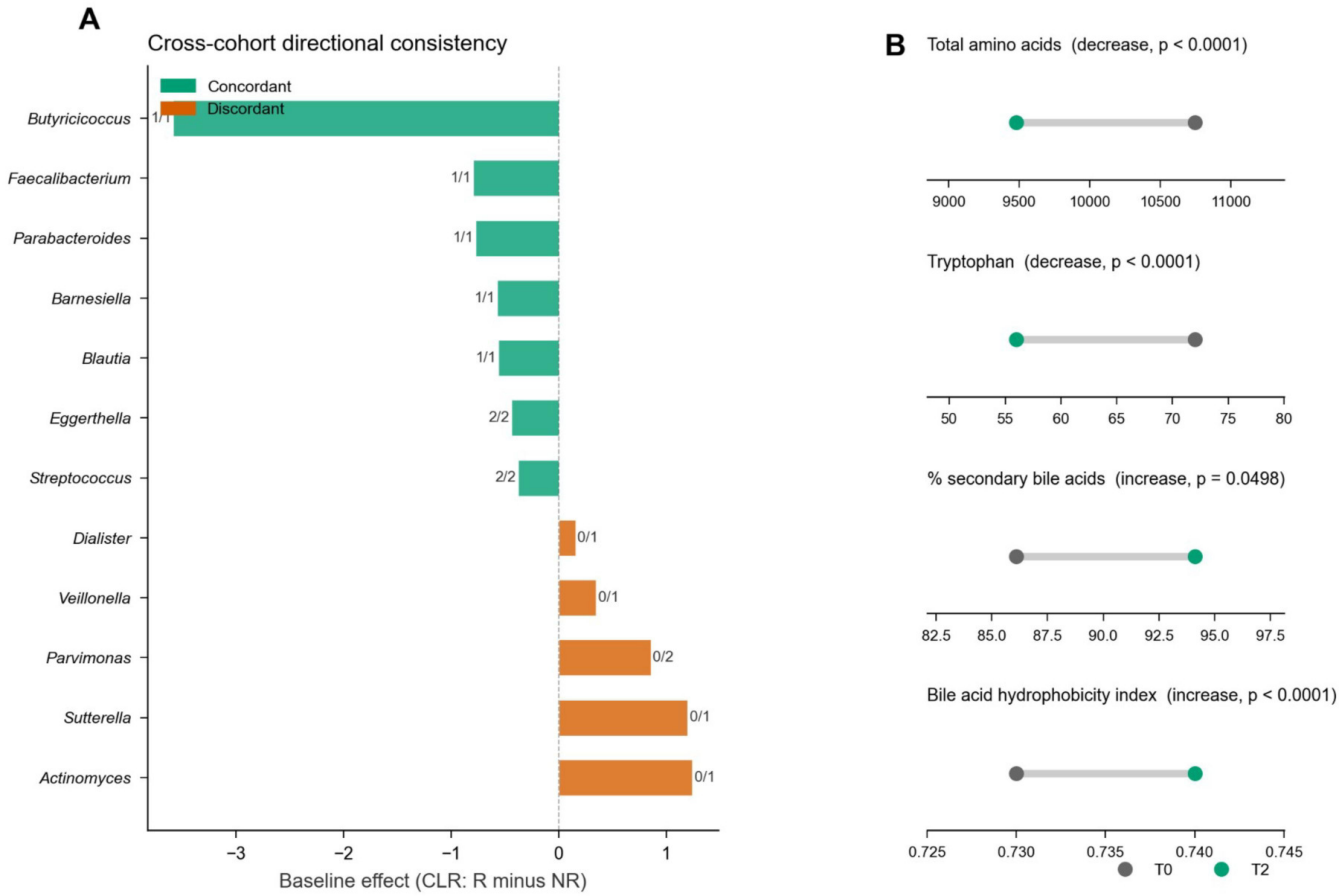
External summary comparison of key genera and measured biochemical context. **(A)** External summary comparison contrasts baseline responder effects in PRJEB14084 (centered log-ratio mean difference, responders minus non-responders) with published differential genera from PRJEB33603 summary tables. Colors indicate concordance after alignment to an inflammation-severity framework. Because the external evidence is based on published summaries without per-participant endpoint extraction, this panel provides supportive comparison only. **(B)** Measured biochemical context in PRJEB14084 during exclusive enteral nutrition shows baseline and end-of-treatment medians for amino-acid and bile-acid readouts. Values are summary-level and interpreted as measured context. Source Data: Source Data 8–9.

External evidence was based on published STAMP summary tables from PRJEB33603, which report differential genera across inflammation severity and high-vs.-low FCP contrasts (17). We compared PRJEB14084 baseline responder effect directions (CLR mean difference: responders minus non-responders) with the reported PRJEB33603 directions after aligning them to an inflammation-severity framework (non-responders in PRJEB14084; severe/high-FCP strata in PRJEB33603). Because participant-level PRJEB33603 endpoints were not re-extracted from the Supplementary Materials, this analysis provides only a summary-level external comparison (Figure 4).

## Results

### EEN-associated remodeling of key genera and fecal biomolecule panels

Among pediatric Crohn's disease participants with paired 16S samples at baseline (T0) and end of EEN (T2; approximately 6 weeks) (*n* = 19), genus-level changes were heterogeneous but showed several consistent directional shifts when summarized as within-subject paired changes (T2 minus T0) (Figure 1B; Source Data 1). Among the taxa with the largest absolute mean changes, *Subdoligranulum* decreased (mean change, −0.037 in relative abundance; 95% CI, −0.071 to −0.003) and *Bacteroides* increased (mean change, +0.023; 95% CI, +0.0004 to +0.046). A taxon annotated as *Guyana* in the source table also decreased (mean change, −0.035; 95% CI, −0.061 to −0.008). Other large shifts included increases in *Holdemanella* (mean change, +0.050; 95% CI, −0.038 to +0.138) and *Collinsella* (mean change, +0.036; 95% CI, −0.018 to +0.090), although the intervals were wider, reflecting substantial inter-individual variability (Figure 1).

To place these findings in biochemical context, we summarized the targeted fecal metabolite panels (HPLC) reported in the source publication (Figures 1C,D; Source Data 2). Compared with baseline (T0), end-of-EEN (T2) values were numerically lower for total amino acids (median, from 10,745 μM to 9,479 μM) and tryptophan (from 72 μM to 56 μM). For bile acids, the hydrophobicity index shifted upward toward the control range (median, 0.73–0.74), and the proportion of secondary bile acids increased (median, 86.1% to 94.1%). Because the Supplementary Material provides only summary statistics, the accompanying *p*-values reflect global Kruskal–Wallis tests across controls and multiple timepoints; the reported Dunn *post hoc* comparisons did not support a significant T0-vs.-T2 difference for these panels. Accordingly, these metabolite results are interpreted as measured biochemical context rather than participant-level mechanistic coupling to taxonomic shifts.

Overall, EEN was associated with heterogeneous but broadly consistent directional genus-level changes across participants, while the HPLC summary panels provide measured biochemical context consistent with partial normalization during induction therapy.

### Response-linked persistence at follow-up

To assess whether a fecal biochemical signature persisted after return to habitual diet, we used follow-up (T3) summary contrasts from 1H-NMR together with follow-up taxonomic distributions (Figure 2; Source Data 3–4). In the NMR discriminant metabolite summary at T3 (contrasts vs. healthy controls with BH-adjusted *p*-values reported in the supplement), responders showed an attenuated signature: only putrescine (adjusted *p* = 0.008) and tryptophan (adjusted *p* = 0.027) remained significant (Figure 2A; Source Data 3). In contrast, non-responders retained a broad discriminant profile, with 12 of 12 reported metabolites remaining significant after adjustment, including amino acids (e.g., valine, leucine, and phenylalanine), propionate, trimethylamine, and polyamines (all reported adjusted *p* ≤ 0.007). These NMR results are used to describe persistence and normalization patterns rather than metabolite direction of change because the supplement reports correlation-based discriminant summaries rather than raw concentrations (Figure 2).

Consistent with this pattern, follow-up (T3) genus-level relative abundances differed between responders (*n* = 5) and non-responders (*n* = 8) for several of the key genera highlighted during EEN (Figure 2B; Source Data 4). For example, non-responders showed higher mean relative abundance of *Holdemanella* at follow-up (0.092 vs. 0.00034 in responders; rank-sum *p* = 0.019) and higher *Collinsella* (0.100 vs. 0.029; *p* = 0.028), consistent with less complete ecological normalization in the non-responder group. These follow-up taxonomic comparisons are descriptive and are intended to align microbial ecology with the response-stratified persistence of the fecal biochemical signature.

Taken together, the follow-up summaries suggest that non-responders may retain a broader fecal biochemical disturbance and distinct genus-level profiles after return to habitual diet, although subgroup sizes are small and the metabolite evidence is summary-only.

### Exploratory baseline discrimination of biochemical response

We next examined whether baseline microbiome composition was associated with biochemical response to EEN (fecal calprotectin reduction of at least 50% at end-of-EEN relative to baseline) using repeated nested cross-validation on baseline samples (*n* = 18) (Figure 3; Source Data 5–7). A logistic regression classifier trained on CLR-transformed OTU features showed modest discrimination with substantial uncertainty (mean ROC AUC, 0.655; 95% CI, 0.450–0.850; mean PR AUC, 0.671; 95% CI, 0.471–0.854) and a mean Brier score of 0.237 (95% CI, 0.202–0.278) (Figure 3A; Source Data 5). In contrast, genus-aggregated features did not support meaningful discrimination in this limited cohort (mean ROC AUC, 0.500; 95% CI, 0.300–0.688). Calibration was summarized from held-out predictions aggregated across repeats (Figure 3B; Source Data 6) (Figure 3).

Collectively, baseline OTU-level CLR features showed only modest discrimination with wide uncertainty and a confidence interval that includes 0.5, consistent with limited and imprecise signal in this small cohort; genus aggregation did not improve discrimination.

For interpretability, we summarized the largest-magnitude genus coefficients from a full-data fit as an illustrative view rather than a claim of coefficient stability (Figure 3C; Source Data 7). Several genera, including *Holdemanella*, *Olsenella*, *Butyricicoccus*, and *Escherichia/Shigella*, had non-zero coefficients and provide a compact hypothesis-generating list for downstream validation.

### External summary comparison and measured biochemical context

We compared PRJEB14084 baseline responder-associated genus effects (CLR mean difference: responders minus non-responders) with published STAMP differential genera from an independent pediatric EEN cohort (PRJEB33603; severity and high/low fecal calprotectin contrasts). Across 12 overlapping genera, 7 showed concordant directions after alignment to an inflammation-severity framework (Figure 4A; Source Data 8). Because the PRJEB33603 evidence is summary-based and endpoints are not fully harmonized, this result should be regarded as supportive comparison only (Figure 4).

To place these findings in biochemical context, we summarized measured stool biomolecule highlights in PRJEB14084 during EEN (HPLC medians from T0 to T2). Total amino acids and tryptophan decreased, whereas bile-acid composition indices, including the proportion of secondary bile acids and the hydrophobicity index, increased (Figure 4B; Source Data 9). Because the metabolite panels were available only in summary form, these findings provide measured biochemical context rather than participant-level microbiome–metabolite coupling tests.

### Integrated schematic overview

Finally, we present a schematic summary of the associations linking baseline microbial signatures, longitudinal remodeling, and the divergent biochemical trajectories observed between responders and non-responders. The schematic is association-focused and is intended to clarify scope and limitations rather than imply causality.

## Discussion

In this secondary analysis of public pediatric EEN cohorts, we observed genus-level remodeling during induction therapy and found that baseline microbiome composition showed a limited but potentially informative association with biochemical response defined by fecal calprotectin reduction. We interpreted these taxonomic patterns alongside reported stool biochemical panels and compared genus-level directions with published summaries from an independent cohort. Interpretation remains cautious throughout because both biochemical and external evidence are available only as summary outputs.

### Principal findings and translational framing

EEN was associated with heterogeneous but broadly consistent directional genus-level changes between baseline and end of treatment, captured as within-subject paired changes (Figure 1; Source Data 1). In parallel, baseline microbiome composition showed a modest association with biochemical response in a repeated nested cross-validation analysis: OTU-level CLR features yielded limited discrimination with wide uncertainty, and calibration was reported explicitly (Figure 3; Source Data 5–6). Taken together, these findings suggest that baseline microbiome features may contain response-related information, but they are not sufficient for clinical use in their current form.

An important interpretive consideration is that many microbiome shifts during EEN may primarily reflect dietary substrate modification (i.e., the EEN formula itself) rather than disease recovery *per se*. In practice, both mechanisms can co-occur: EEN changes nutrient availability and gut ecology, while reductions in inflammation can secondarily reshape the microbiome. With the available public data, we cannot disentangle these components definitively, and we therefore interpret longitudinal changes as treatment-associated remodeling rather than as causal mediators of mucosal healing.

Follow-up contrasts further suggested response-linked persistence of a fecal biochemical disturbance after return to habitual diet. Responders showed attenuation to two significant discriminant metabolites at T3, whereas non-responders retained a broader discriminant profile that included amino acids, propionate, trimethylamine, and polyamines (Figure 2; Source Data 3). Although these NMR outputs are reported as discriminant or correlation summaries rather than raw concentrations, the persistence pattern provides a qualitative link between clinical response and molecular context. In the external summary comparison, most overlapping genera showed concordant directions after alignment to an inflammation-severity framework (Figure 4; Source Data 8). This provides limited support for biological consistency, but does not substitute for patient-level validation.

### Biochemical context and interpretation boundaries

Mechanistically, our findings are most plausibly interpreted as a diet-driven ecological perturbation that co-occurs with shifts in the fecal biomolecular milieu. The targeted HPLC summary panels during EEN indicate decreases in total amino acids and tryptophan and increases in bile-acid composition indices (Figures 1, 4; Source Data 2 and 9). These biomolecule classes are plausible mediators or markers of host–microbe interactions in the inflamed gut, including nutrient availability, microbial competition, and bile-acid signaling. However, because the HPLC panels are available only as summary statistics (medians and IQRs with global tests), we do not test participant-level microbiome–metabolite coupling, mediation, or causality. Similarly, the follow-up NMR summary is interpreted as persistence or normalization relative to controls rather than as a quantitative metabolite trajectory.

Prior work links gut microbial metabolites (including bile acids, short-chain fatty acids, and tryptophan-derived indoles) to barrier function and immune signaling in intestinal inflammation (21–30). In our setting, we treat the fecal biochemical readouts as measured context that supports biological plausibility rather than as evidence of participant-level microbiome–metabolite coupling. This framing keeps the mechanistic narrative plausible while avoiding causal claims that are not supported by metabolite data available only in summary form.

Recent pediatric dietary-therapy literature further supports the potential relevance of tryptophan-pathway readouts as candidate biochemical markers of response in pediatric Crohn's disease (31), and recent reviews synthesize diet–microbiota interactions in inflammatory bowel disease from a clinical and nutritional perspective (32). More broadly, recent syntheses of therapeutic options and predictive biomarkers in inflammatory bowel disease emphasize the ongoing need for accessible, non-invasive response markers to support precision treatment selection (33).

### Implications for response stratification and future validation

From an applied perspective, these findings support prospective evaluation in cohorts where microbiome profiles and standardized biochemical endpoints are collected at adequate sample size. Calibration remains important, but clinical utility cannot be inferred from the present analysis. In future work, a prespecified modeling and evaluation plan would help reduce optimism in small datasets. If participant-level metabolite concentrations become available, microbiome–biochemical coupling could be examined while maintaining conservative language around causal inference.

### Limitations

Several limitations are central to interpretation. First, the microbiome analyses are based on 16S-derived composition and genus-level aggregation; the results are compositional and cannot directly quantify absolute biomass or strain-level functions. Second, the discovery cohort sample sizes are small (*n* = 18 for baseline discrimination and *n* = 19 for paired baseline-to-end-of-EEN 16S), resulting in wide uncertainty and limited scope for subgroup analyses, including follow-up response-stratified comparisons (T3 16S: responders *n* = 5; non-responders *n* = 8). Third, the HPLC metabolite panels were available only in summary form, and the NMR outputs are discriminant summaries rather than raw concentrations; consequently, participant-level multi-omics integration and causal claims are not supported. Fourth, the external evidence is based on published STAMP summary tables from PRJEB33603; we did not re-estimate effect sizes using participant-level external endpoints, and this component should therefore be interpreted as supportive summary comparison rather than validation. These limitations define the boundary of defensible claims. Additionally, the baseline association model performance is not sufficient to support clinical deployment, and is presented only to quantify uncertainty and support hypothesis generation in the available public data setting.

## Conclusions

Using publicly available pediatric EEN cohorts, we found that baseline microbiome features showed an exploratory association with fecal calprotectin response and that EEN was associated with genus-level remodeling. Response status was also associated with different follow-up biochemical persistence patterns in NMR summary outputs, and an independent cohort provided a limited summary-level comparison at the genus level. These findings support hypothesis generation for non-invasive response stratification in pediatric Crohn's disease, but require prospective confirmation.

## Data Availability

The datasets analyzed in this study are publicly available in the European Nucleotide Archive under accession numbers PRJEB14084 and PRJEB33603. Additional summary-level metabolite and differential-taxa results are available in the supplementary materials of the original source publications. The Source Data files and analysis code supporting this article are archived in Zenodo at https://doi.org/10.5281/zenodo.18815000; the code is also available at https://github.com/Zhenghongwei11/pediatric-cd-een-microbiome-response.
